# Green tea and quercetin sensitize PC-3 xenograft prostate tumors to docetaxel chemotherapy

**DOI:** 10.1186/s13046-016-0351-x

**Published:** 2016-05-06

**Authors:** Piwen Wang, Susanne M. Henning, Clara E. Magyar, Yahya Elshimali, David Heber, Jaydutt V. Vadgama

**Affiliations:** Division of Cancer Research and Training, Charles R. Drew University of Medicine and Science, Los Angeles, CA 90059 USA; Center for Human Nutrition, Department of Medicine, David Geffen School of Medicine, University of California, Los Angeles, CA 90095 USA; Department of Pathology, David Geffen School of Medicine, University of California, Los Angeles, CA 90095 USA; Department of Medicine, David Geffen School of Medicine, University of California, Los Angeles, CA 90095 USA

**Keywords:** Green tea polyphenol, Quercetin, Docetaxel, Prostate cancer, Combination

## Abstract

**Background:**

Chemotherapy with docetaxel (Doc) remains the standard treatment for metastatic and castration-resistance prostate cancer (CRPC). However, the clinical success of Doc is limited by its chemoresistance and side effects. This study investigated whether natural products green tea (GT) and quercetin (Q) enhance the therapeutic efficacy of Doc in CRPC in mouse models.

**Methods:**

Male severe combined immunodeficiency (SCID) mice (*n* = 10 per group) were inoculated with androgen-independent prostate cancer PC-3 cells subcutaneously. When tumors were established the intervention started. Mice were administered with GT + Q, Doc 5 mg/kg (LD), GT + Q + LD Doc, Doc 10 mg/kg (HD) or control. The concentration of GT polyphenols in brewed tea administered as drinking water was 0.07 % and Q was supplemented in diet at 0.4 %. Doc was intravenously injected weekly for 4 weeks, GT and Q given throughout the study.

**Results:**

GT + Q or LD Doc slightly inhibited tumor growth compared to control. However, the combination of GT and Q with LD Doc significantly enhanced the potency of Doc 2-fold and reduced tumor growth by 62 % compared to LD Doc in 7-weeks intervention. A decrease of Ki67 and increase of cleaved caspase 7 were observed in tumors by the mixture, along with lowered blood concentrations of growth factors like VEGF and EGF. The mixture significantly elevated the levels of tumor suppressor mir15a and mir330 in tumor tissues. An increased risk of liver toxicity was only observed with HD Doc treatment.

**Conclusions:**

These results provide a promising regimen to enhance the therapeutic effect of Doc in a less toxic manner.

## Background

Prostate cancer is the most commonly diagnosed male malignancy and the second-leading cause of cancer death among men in the United States [1]. Chemotherapy with docetaxel (Doc) remains the standard treatment for metastatic and castration-resistance prostate cancer (CRPC) and a backbone in current drug development [2]. Doc belongs to the family of taxanes and it is a microtubule stabilizer inducing mitotic arrest and ultimately cell apoptosis [3]. However, the development of chemoresistance to Doc is observed in most patients associated with the overexpression of anti-apoptotic gene Bcl-2 and activation of nuclear factor-kappa B (NFκB) and PI3K/Akt pathways, which limits its clinical success [2, 4]. The median progression-free survival with Doc treatment remains around 6 months and overall survival is less than 2 years [2]. In addition, there are severe side effects associated with Doc treatment including the suppression of bone marrow function leading to immunodysfunction and anemia [5]. Clearly, it holds high clinical significance to enhance the efficacy of Doc at lower doses in a less-toxic manner and to reduce its side effects.

Green tea (GT) is produced from the leaves of the plant *Camellia sinensis*. The anti-cancer activities of GT have been demonstrated in several cancers including prostate, mammary gland, colon, pancreas, liver, esophagus and liver cancer [6, 7]. The major bioactive components of GT are GT polyphenols (GTPs), mainly including (-)-epigallocatechin (EGC), (-)-epigallocatechin-3-gallate (EGCG), (-)-epicatechin (EC), and (-)-epicatechin-3-gallate (ECG), with EGCG as the most abundant and most bioactive component [6]. Quercetin (Q) is a flavonoid widely found in vegetables and fruits particularly in onions, apples, and red wine. We were able to demonstrate in vitro that the combination of GT and Q with Doc synergistically enhanced the anti-proliferative effect in androgen-independent PC-3 and LAPC-4-AI cells [8]. The combined effect was associated with increased apoptosis and cell cycle arrest in both cell lines [8]. Apoptosis through the mitochondrial (intrinsic) pathway can be initiated by chemotherapy or other stimuli such as reduced cytokines/growth factors. The pro-apoptotic BCL2 family proteins like Bax, Bak and Bcl-2-associated death promotor (Bad) are important mediators of these signals. Dephosphorylated Bad forms a heterodimer with the anti-apoptotic BCL2 family members Bcl-2 and Bcl-xL, which allows Bax and Bak to aggregate and initiate apoptosis [8]. The subsequent activation of caspases including caspase 3 and 7 leads to the cleavage of their substrate poly (ADP-ribose) polymerase 1 (PARP1), which is a hallmark of apoptosis [9]. Apoptosis through mitochondria can be inhibited by survival signals, such as growth factors and cytokines, through activation of anti-apoptotic pathways such as the NFκB pathway. In cytoplasm, NFκB is bound and inhibited by the inhibitor of NFκB protein (IκB). Once IκB is phosphorylated, NFκB will be released and translocated to nucleus where it induces the expression of target genes to promote cell proliferation and survival [10].

The multiple-targeting activities of GT and Q in anti-carcinogenesis, particularly by targeting the NFκB and pro-apoptotic pathways, make them ideal candidates to be combined with Doc to enhance the therapeutic effect in a less-toxic manner. In in vitro studies we have observed a synergistic anti-proliferative effect by combining GT and Q with Doc [8]. The present study was performed to confirm the combined effect of the mixture in vivo in a xenograft mouse model. This study provides evidence that the combination of GT, Q and Doc enhances the chemotherapeutic activity of Doc and encourages future use in clinical practice in treatment of CRPC with enhanced drug efficacy and reduced side effects.

## Methods

### Preparation of GT water, Q diet and Doc solution

GT was prepared by brewing one tea bag (Celestial Seasonings, Boulder, CO) in 240 mL of boiling water for 5 min. The GT water contained 0.07 % of the major GTPs including (mg/L): EGC 204 ± 4, EGCG 388 ± 12, EC 44 ± 2, ECG 64 ± 7, and catechin 7 ± 1. GT was freshly prepared three times per week on Monday, Wednesday and Friday and administered as drinking water *ad libitum*. Q (Sigma-Aldrich, St Louis, MO) was supplemented in AIN-93G diet at a concentration of 0.4 % by Dyets Inc. (Bethlehem, PA). Docetaxel Injection (Besse Medical, West Chester, OH) was diluted in sodium chloride for tail vein injection.

### Animal study

All procedures carried out in mice were approved by the Charles R. Drew University of Medicine and Science Institutional Animal Care and Use Committee. Male severe combined immunodeficiency (SCID) mice (Charles River Laboratories) of 5–7 weeks old were acclimated to a sterilized AIN-93G diet (Dyets Inc.) and water for seven days. Mice were inoculated subcutaneously with 5x10^5^ androgen-independent PC-3 human prostate cancer cells. The PC-3 cell line (ATCC® CRL-1435™) was purchased from American Type Culture Collection (Manassas, VA), where the cells were authenticated by STR DNA profiling and tested as mycoplasma free. PC-3 cells were maintained in RPMI 1640 medium supplemented with 10 % (v:v) of fetal bovine serum, 100 IU/mL of penicillin and 100 μg/mL of streptomycin at 37 °C in a 5 % CO2 incubator, and were tested periodically for mycoplasma contamination. The passage of the PC-3 cell line used for the inoculation was <10. Four weeks after inoculation when tumors reached a volume of 50–80 mm^3^ the intervention treatment started. Mice were randomly assigned to one of the five groups (*n* = 10 per group) including: 1) control, receiving AIN-93G diet + water + vehicle (sodium chloride); 2) GT + Q, receiving Q diet + GT + vehicle; 3) low dose (LD) Doc, receiving AIN-93G diet + water + 5 mg/kg Doc; 4) GT + Q + LD Doc, receiving Q diet + GT + 5 mg/kg Doc ; and 5) high dose (HD) Doc, receiving AIN-93G diet + water + 10 mg/kg Doc [11]. Doc was administered once a week for 4 weeks, GT and Q throughout the study. Tumor size was measured once a week with calipers. Tumor volume was calculated using the formula: length x width x height x 0.5236 [12]. Mouse body weight was measured once a week. Mice in the control and GT + Q groups were sacrificed when they had received 5-weeks intervention treatment in observance of the institutional guideline on tumor size. Other groups were sacrificed 2 weeks later.

### Apoptosis signaling antibody array assay

Eight tumor samples were randomly selected from each group for all the following molecular analyses in tumors. A slide-based antibody array was used for simultaneous detection of 19 important signaling molecules involved in stress response and apoptosis, using a PathScan Stress and Apoptosis Signaling Antibody Array kit (Cell Signaling Technology, Danvers, MA) following the manufacturer’s instructions. Each of these molecules was detected in duplicate on the same array arranged as shown in Fig. 2c, and the identity of each numbered molecule listed in Fig. 2d. Briefly, total protein was extracted from tumor tissues using RIPA buffer (Santa Cruz Technology, CA), and diluted to 0.5 mg/mL in Array Diluent Buffer provided by the kit. The protein samples were incubated with the array antibodies overnight. A Detection Antibody Cocktail was added to the samples, followed by the addition of HRP-linked Streptavidin and substrate. Protein was visualized using a ChemiDoc XRS chemiluminescence detection and imaging system (Bio-Rad Laboratories, Irvine, CA). The density of the spots were quantitated using Quantity One software (Bio-Rad Laboratories) and normalized to the α-tubulin levels. Each sample was done in duplicate.

### Western blot analysis of protein markers of apoptosis and angiogenesis

Total protein was extracted from tumor tissues using RIPA lysis buffer (Santa Cruz, CA). The Western blot procedure was described previously [13]. Briefly, 50 μg of protein was separated on a 4–12 % Bis-Tris gel (Invitrogen, Carlsbad, CA), electrotransferred to nitrocellulose membranes and blocked in Tris-buffered saline with 0.1 % Tween 20 and 5 % nonfat milk for 1 h at room temperature. Membranes were incubated with primary antibodies for the detection of Bax (sc-493), Bcl-2 (sc-509), and vascular endothelial growth factor (VEGF, sc-152), respectively. GAPDH protein was used as loading control. Images were visualized and quantified using a ChemiDoc XRS chemiluminescence detection system (Bio-Rad Laboratories). Each sample was done in duplicate.

### Tissue microarray and immunohistochemical analysis of proliferation

A section of each tumor was fixed in 10 % phosphate buffered formalin and paraffin-embedded for tissue microarray and immunohistochemical detection. The procedures for tissue array assembling and immunohistochemical detection were described before [14]. Briefly, a total of 6 cylindrical cores 1.0 mm in diameter were transferred from each donor block to two new paraffin array blocks. Four-micron sections were cut from each array for staining. The slides were prepared for immunohistochemistry by deparaffinizing and dehydrating, followed by an alcohol series and washing in PBS. All samples were incubated in 3 % H_2_O_2_ to eliminate endogenous peroxidase activity, and antigens were retrieved by boiling the slides in a microwave oven. After blocking with goat serum the slides were incubated with monoclonal Ki67 antibody (DAKO North America Inc., Carpineteria, CA). The slides were counterstained with hematoxylin. Slides were digitized on a ScanScope AT (Aperio Technologies, Inc., Vista, CA) and morphometric analysis performed with Definiens’ Tissue Studio (Definiens Inc., Parsippany, NJ) to determine the percentage of Ki67 positive cells in a non-biased method.

### Measurement of growth factors in blood

Several growth factors play important roles in tumor cell proliferation and angiogenesis. The blood levels of growth factors were measured using a Human Growth Factor ELISA Strip II kit (Signosis, inc., Santa Clara, CA). Eight blood samples were randomly selected from each group for this analysis. The kit allows a simultaneous quantification of 8 growth factors, including VEGF, epidermal growth factor (EGF), platelet-derived growth factor (PDGF)-BB, nerve growth factor (NGF)-β, stem cell factor (SCF), tumor necrosis factor (TNF)-α, fibroblast growth factor (FGF)-β, and transforming growth factor (TGF)-β. Briefly, serum samples were diluted by 1:10 and 100 μl was added to each well of the 8-wells strip coated with different antibodies. After 1 h incubation the wells were aspirated and washed. A biotin-labeled antibody was added to the samples followed by streptavidin-HRP conjugate. The luminescence was measured after adding a HRP substrate solution. The expression level of these growth factors is proportional to the luminescent intensity.

### qRT-PCR analysis of microRNA expression

Total microRNA (miRNA) was extracted from tumor tissues using a miRNeasy mini kit (Qiagen), and reversely transcribed to cDNA using miScript II RT kit (Qiagen). Specific primers were provided by miScript Primer Assays for miR-15a (MS00003178), miR-330 (MS00009450), and miR-19b (MS00031584). The quantitative real-time (qRT)-PCR procedure was described previously [15]. Briefly, a miScript SYBR Green PCR kit (Qiagen) was used for the reaction. The 25 μL final volume of reaction contained 12.5 μL of 2x QuantiTect SYBR Green PCR Master Mix, 2.5 μL of 10x miScript Universal Primer, 2.5 μL of 10x miScript Primer Assay, 2.5 μL of template cDNA, and RNase-free water. The reaction mixture was incubated at 95 °C for 15 min, followed by 40 cycles of 94 °C for 15 s, 55 °C for 30 s, and 70 °C for 30 s. Mature miRNA expression was calculated using the 2^-ΔΔCt^ method in normalization to human RNU6-2 snRNA (MS00033740). Each sample was done in triplicate.

### Blood ALT and AST measurement

Side effect is one of the major issues that limit the dosage and efficacy of most chemotherapy drugs. The damage on liver is frequently found with Doc treatment and is an indicator of Doc toxicity [16]. The blood levels of liver enzymes like alanine transaminase (ALT) and aspartate transaminase (AST) are commonly used as markers of liver damage. The possibility of treatment as well as cancer related liver damage was evaluated by both blood enzyme and liver pathological evaluations in the present study. Blood samples were collected at mouse sacrificing and serum separated. Eight blood samples were randomly selected from each group for this analysis. The blood ALT and AST levels were determined by measuring their colorimetric products pyruvate and glutamate, respectively, using assay kits (Sigma-Aldrich). 20 ul of serum was used for each reaction and each sample was done in triplicate.

### Liver pathological examination

A section of liver tissue was fixed in 10 % phosphate buffered formalin and paraffin-embedded. Eight liver samples were randomly selected from each group for pathological examination. Slides were cut and stained with H&E. The pathologist Dr. Elshimali did the liver pathological examination in a blind manner. The liver condition was graded based on the degree of inflammation, piecemeal or bridging necrosis with the following criteria: Grade 0: no/minimal inflammation; Grade 1: portal inflammation or lobular inflammation without necrosis; Grade 2: mild periportal inflammation and piecemeal necrosis or focal hepatocellular necrosis; Grade 3: moderate periportal inflammation and piecemeal necrosis or severe focal cell damage; Grade 4: severe periportal inflammation and piecemeal necrosis or bridging necrosis.

### Statistical analysis

Data were expressed as mean ± standard deviation (SD) or mean ± standard error (SE). Comparison of means was performed by one-way analysis of variance (ANOVA) with Tukey’s posttest for paired comparison. Differences were considered significant if *P* < 0.05. SPSS (Version 20, Chicago, IL) was used for all statistical analysis.

## Results

### Enhanced inhibition of PC-3 xenograft tumor growth

**Fig. 1:**
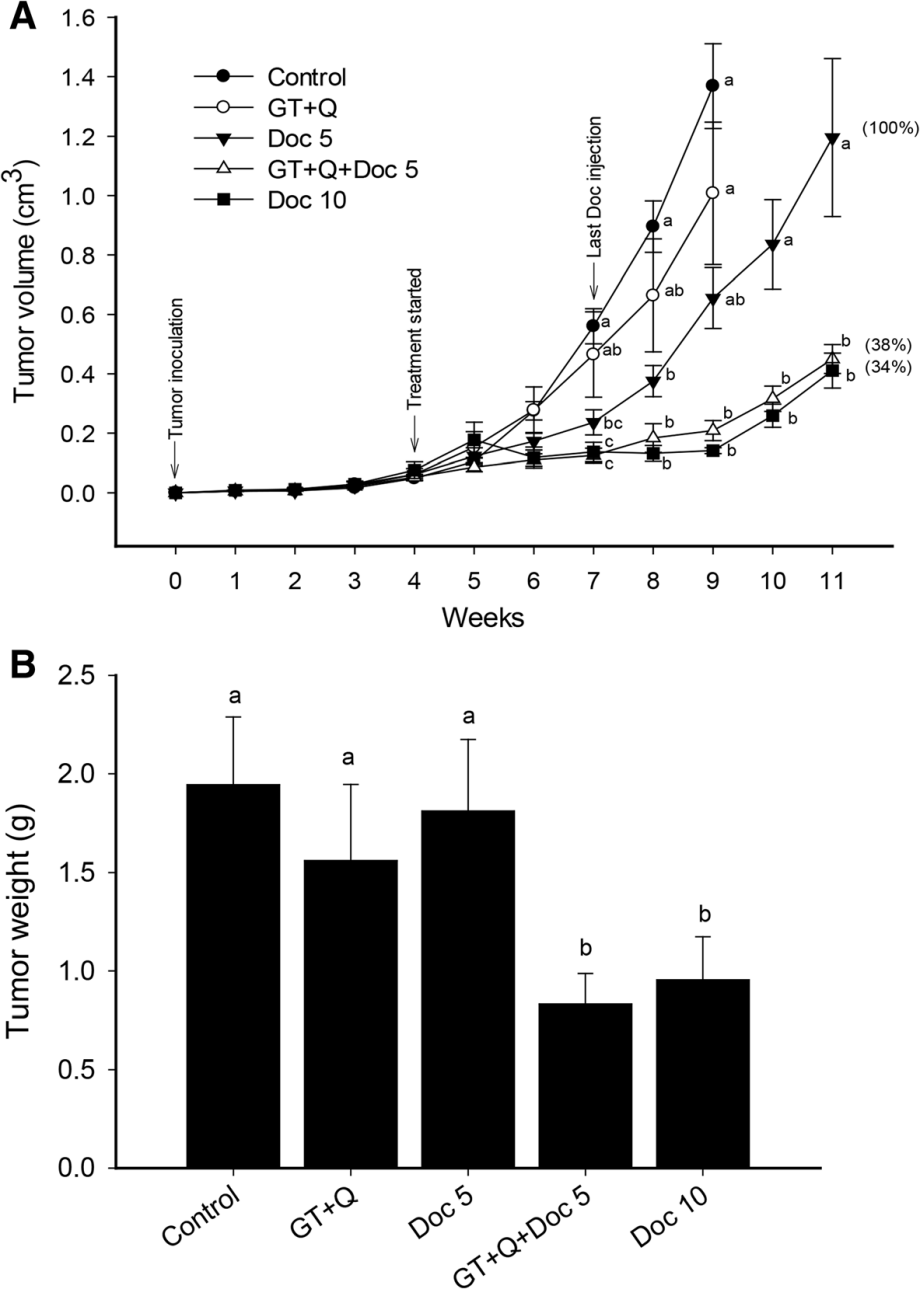
GT and Q in combination with Doc enhanced the inhibition of PC-3 xenograft tumor growth in SCID mice. Male SCID mice were inoculated subcutaneously with 5x10^5^ androgen-independent PC-3 prostate tumor cells. The intervention treatments started 4 weeks later when tumors were established. Doc was injected through tail vein once a week for 4 weeks. GT and Q were administered throughout the study. Tumor size was measured using caliper and volume calculated using the formula: length x width x height x 0.5236. Data are presented as mean ± SE for tumor volume (**a**) or mean ± SD for tumor weight (**b**). *N* = 10 per group. GT, green tea; Q, quercetin; Doc 5, docetaxel at 5 mg/kg iv; Doc 10, docetaxel at 10 mg/kg iv. Different letters at each time points represent significant difference between treatments (*P* < 0.05)

Different profiles of tumor growth were observed between treatments. Only treatments with the HD Doc and combination of GT + Q + LD Doc significantly decreased tumor growth throughout the intervention (Fig. 1a). LD Doc treatment initially induced a significant inhibition of tumor growth but after week 8 tumor growth accelerated (Fig. 1a). GT + Q treatment did not inhibit tumor growth significantly compared to control (Fig. 1a). In summary at week 9, the tumor growth was inhibited by 26 % (GT + Q), 52 % (LD Doc), and 85 % (GT + Q + LD Doc) compared to control. At week 11, the mixture of all three chemicals reduced the tumor growth by 62 % compared to LD Doc alone. The combined effect of the mixture was comparable to that by a two-fold higher dose of Doc (HD Doc) (Fig. 1a). A consistent pattern was observed between tumor weight and tumor volume (Fig. 1b).

### Increased tumor cell apoptosis

**Fig. 2:**
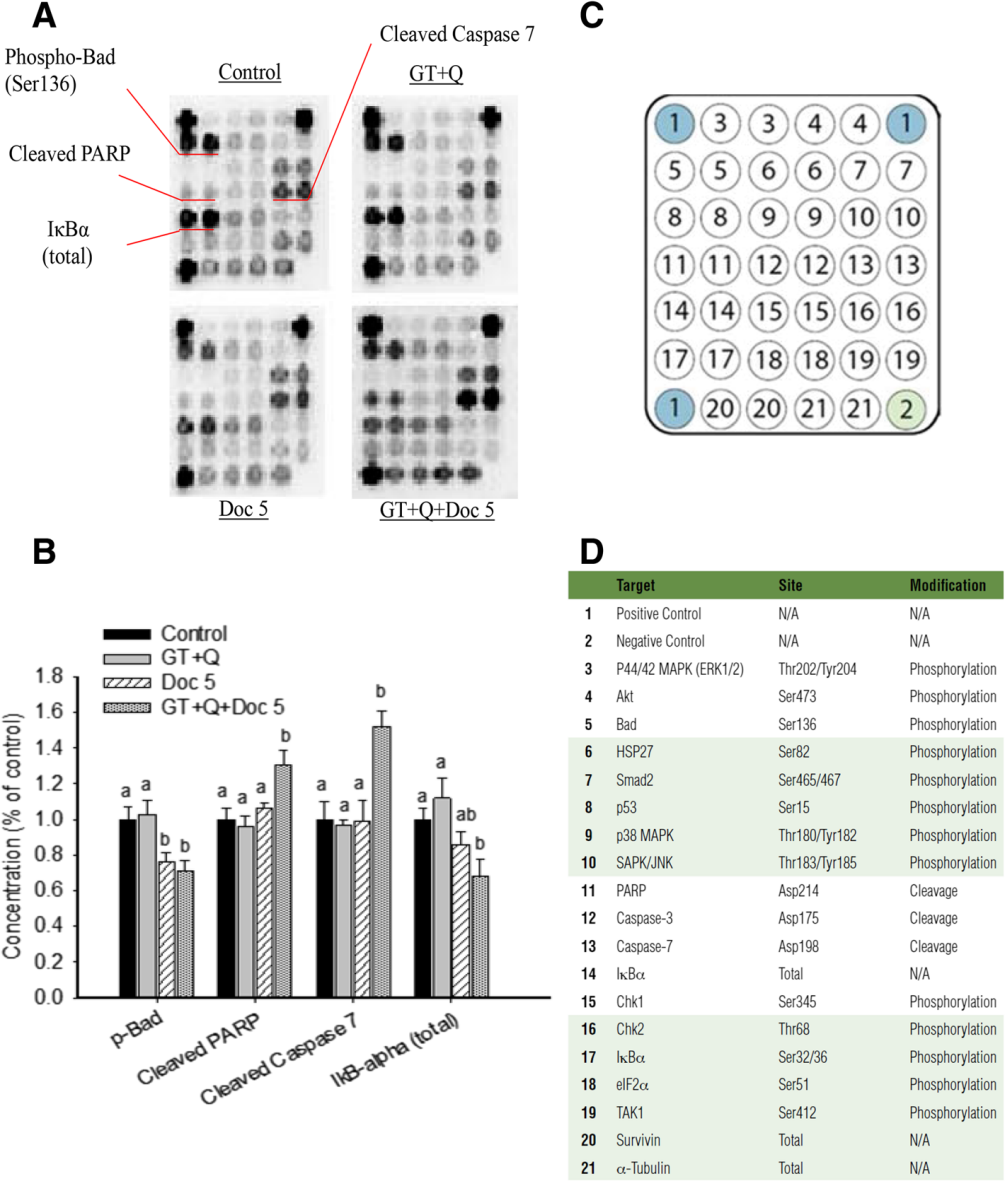
Modulation of apoptosis related signaling proteins using antibody array assay. Eight tumor samples were randomly selected from each group for this analysis. Total protein was extracted from the tumor tissues. A slide-based antibody array was used for simultaneous detection of 19 signaling molecules (**c** and **d**) involved in stress response and apoptosis using a PathScan Stress and Apoptosis Signaling Array kit. Each protein was arranged in duplicate. The names of the proteins with significant changes in concentration were indicated on the images (**a**) and their concentrations quantified (**b**). Data are presented as mean ± SD. GT, green tea; Q, quercetin; Doc 5, docetaxel at 5 mg/kg iv. Columns with different letters represent significant difference between treatments (*P* < 0.05)

**Fig. 3:**
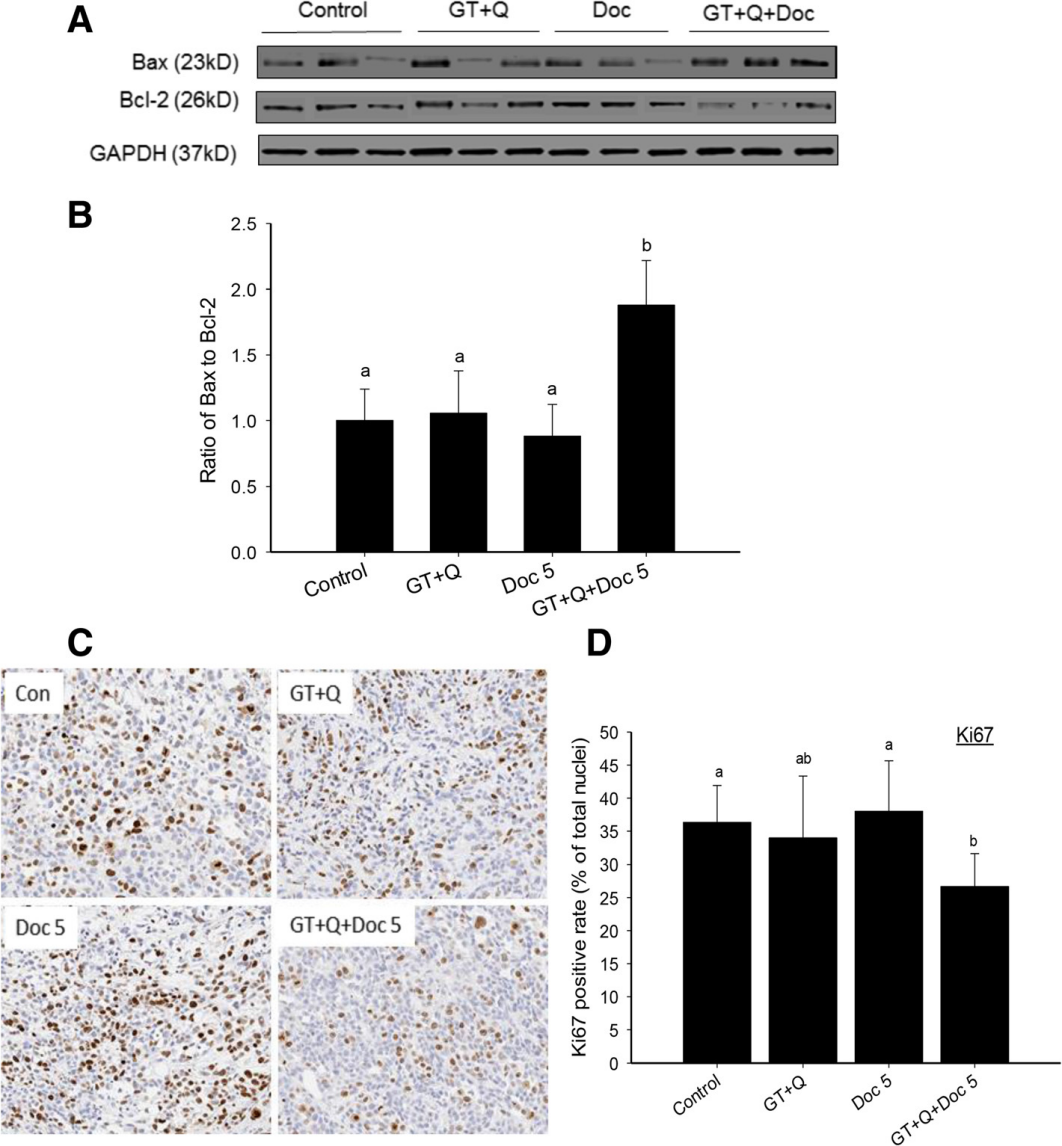
Increased Bax/Bcl-2 ratio and decreased Ki67 protein expression by GT, Q and Doc in combination. Eight tumor samples were randomly selected from each group for this analysis. The protein expression of Bax and Bcl-2 in tumor tissues was detected by Western blot. Three representative protein images from each group were demonstrated (**a**). The ratios of Bax to Bcl-2 are presented as mean ± SD (**b**). A section of tumor tissue was formalin-fixed and paraffin-embedded for tissue microarray and immunohistochemical detection. Slides were cut from the arrays and incubated with primary antibodies for detection of Ki67 (**c**). The slides were counterstained with hematoxylin. Nuclei were stained in blue and Ki67 in brown color. The positive rates of Ki67 nuclear staining are presented as mean ± SD (**d**). GT, green tea; Q, quercetin; Doc 5, docetaxel at 5 mg/kg iv. Columns with different letters represent significant difference between treatments (*P* < 0.05)

A protein array analysis was performed to determine the effect of different treatments on apoptotic signaling pathways, including molecules involved in the mitochondrial (intrinsic) pathway such as Bad, caspases, and PARP, and NFκB pathway. The treatment with GT + Q was not significantly effective to modulate these pathways in the tumor tissues compared to control (Fig. 2a and b). Both LD Doc alone and its combination with GT and Q significantly decreased the phosphorylation of Bad protein compared to control. However, a significant increase of cleaved PARP (by 30 % compared to control) and cleaved caspase 7 (by 52 %) and decrease of IκBα (by 32 %) were only achieved by the combination of all three chemicals (Fig. 2a and b). Western blot analysis of Bax and Bcl-2 protein expression in tumor tissues found no significant difference in Bax/Bcl-2 ratio with the treatment of GT + Q or LD Doc alone compared to control. However, the combination of GT, Q and LD Doc significantly increased the ratio of Bax/Bcl-2 compared to other treatments (Fig. 3a and b).

### Decreased tumor cell proliferation

Tumor cell proliferation under different treatments was evaluated by the levels of the proliferation marker nuclear Ki67 protein in tumor tissues using tissue microarray and immunohistochemical analysis. There was no significant changes in tumor Ki67 levels in GT + Q or LD Doc alone groups compared to control. However, the combination of GT and Q with LD Doc significantly decreased the Ki67 levels compared to both control and LD Doc (Fig. 3c and d).

### Downregulation of tumor growth factors

**Fig. 4:**
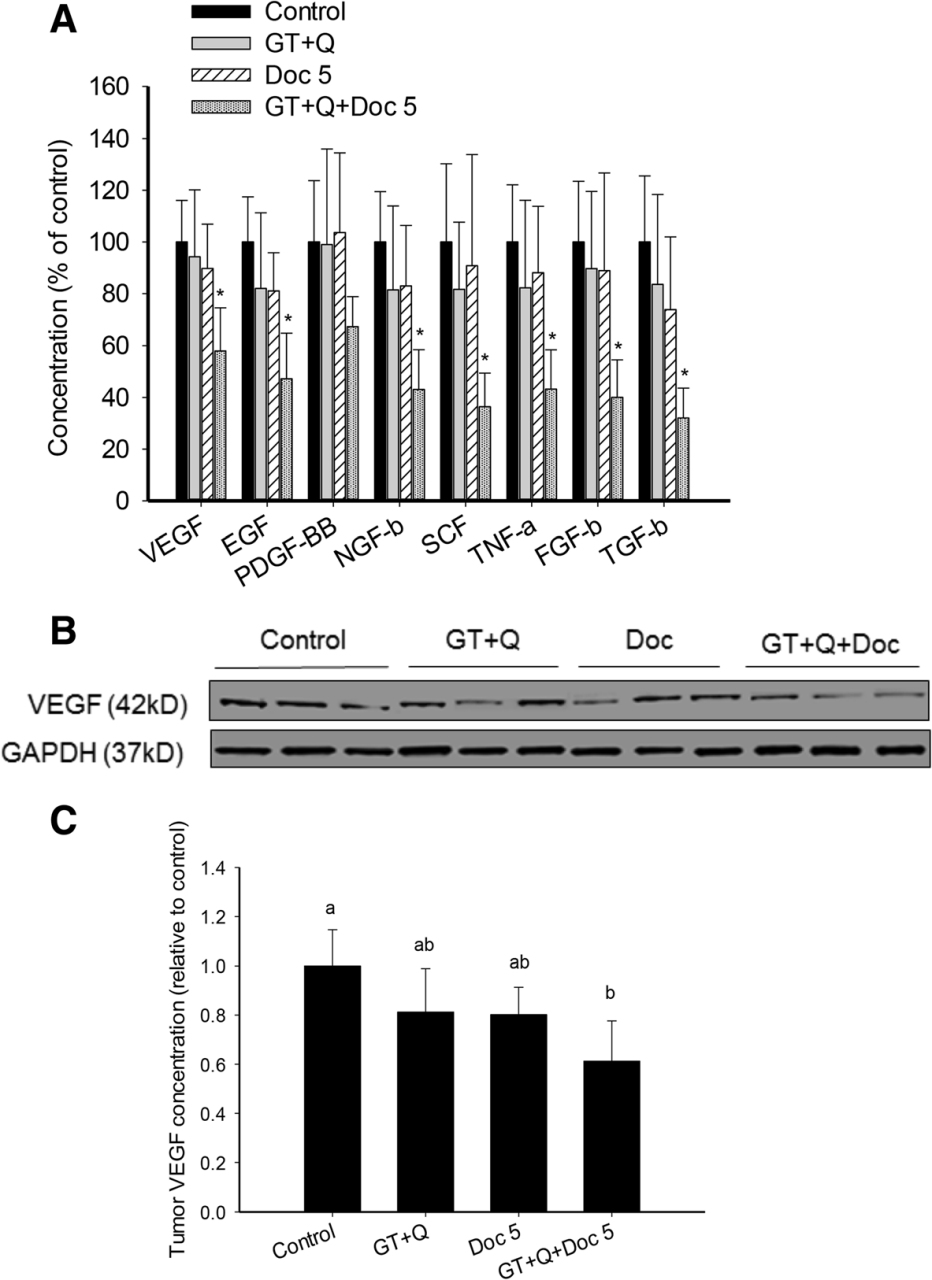
Decreased blood concentrations of tumor growth factors and tumor concentrations of VEGF expression by combining GT and Q with Doc. Blood samples were collected from each mouse at sacrificing and serum separated. Eight blood samples were randomly selected from each group for this analysis. The levels of 8 growth factors in blood were simultaneously measured using a Human Growth Factor ELISA Strip II kit. Data are presented as mean ± SD (**a**). Eight tumor samples were randomly selected from each group for Western blot analysis of VEGF protein expression. Three representative protein images from each group were demonstrated (**b**). The relative concentrations of VEGF in tumor tissues are presented as mean ± SD (**c**). GT, green tea; Q, quercetin; Doc 5, docetaxel at 5 mg/kg iv. Columns with different letters represent significant difference between treatments (*P* < 0.05)

The tumor stimulating activities of the eight analyzed growth factors, including VEGF, EGF, PDGF-BB, NGF-β, SCF, TNF-α, FGF-β, and TGF-β, have been demonstrated in many studies [17–19]. Several of them such as EGF and TGF-β were found to be involved in the chemoresistance to Doc [20, 21]. The capacity of GT, Q and Doc in reducing the expression of these growth factors were analyzed in eight tumor tissues from each group. There was a slight but nonsignificant decrease in the blood levels of these growth factors with GT + Q or LD Doc treatment (Fig. 4a). However, the combination of GT, Q and LD Doc significantly decreased the levels of most growth factors including VEGF, EGF, NGF-β, SCF, TNF-α, FGF-β, and TGF-β, by 40 to 70 % compared to control, with a nonsignificant reduction of PDGF-BB level (Fig. 4a). To confirm the correlation between the blood and tissue concentrations of these growth factors, the expression of VEGF in tumor tissues was analyzed by Western blot. The pattern of VEGF expression in tumor tissues was consistent with that in blood under different treatments (Fig. 4b and c).

### Modulation of miRNA expression

**Fig. 5:**
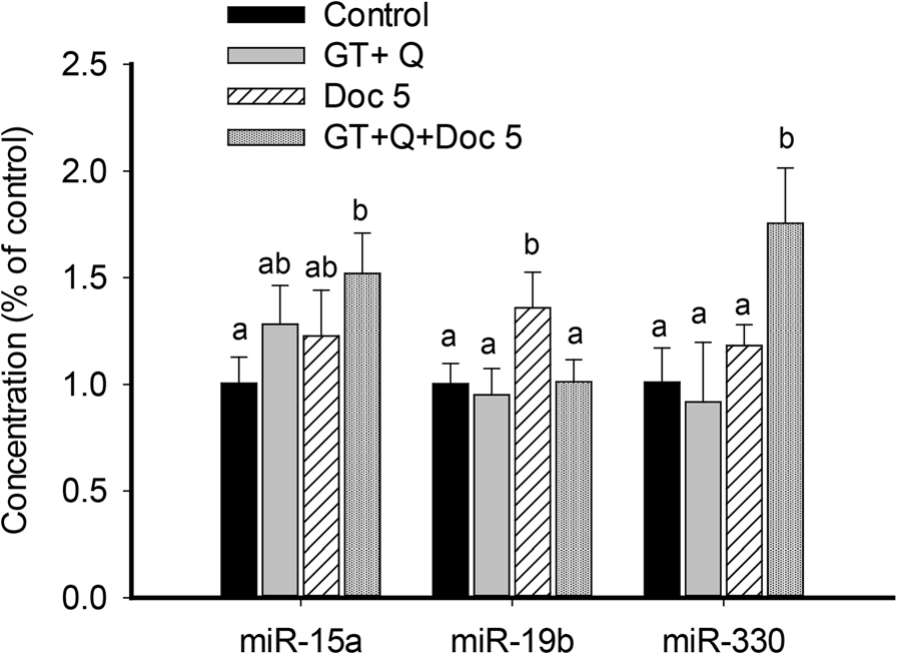
Modulation of miRNA expression. Eight tumor samples were randomly selected from each group for this analysis. Total miRNA was extracted from tumor tissues, reversely transcribed to cDNA and quantified using quantitative real-time PCR. Mature miRNA expression was calculated using the 2^-ΔΔCt^ method in normalization to human RNU6-2 snRNA. Each sample was done in triplicate. Data are presented as mean ± SD. GT, green tea; Q, quercetin; Doc 5, docetaxel at 5 mg/kg iv. Columns with different letters represent significant difference between treatments (*P* < 0.05)

Several phytochemicals including EGCG and Q have demonstrated the ability to modulate the expression of miRNA, a class of small non-coding RNAs that interact with target mRNA to negatively regulate the gene expression at the post-transcriptional level [22–24]. The importance of miRNAs in carcinogenesis has been demonstrated by many studies. MiRNAs have been found to be involved in tumor growth, invasion, angiogenesis, and immune evasion, and they are potential therapeutic targets [25]. To investigate whether miRNAs are responsive to the combination treatment of GT, Q and Doc, we selected three candidate miRNAs that have been shown to be involved in prostate cancer, including two tumor suppressor miR-15a and miR-330 and an oncomiR miR-19b. A different profile of miRNA expression was observed in tumor tissues with GT + Q, Doc or their combination treatment. Both GT + Q and LD Doc alone demonstrated a non-significant trend to increase the expression of miR-15a compared to control, while their combination significantly elevated the level of miR-15a (Fig. 5). Similarly, the mixture of all three chemicals significantly increased miR-330 expression compared to other treatments. A significantly increased expression of the oncogenic miR-19b was observed with LD Doc alone compared to control, however, the level of miR-19b was reduced to the control level when combining LD Doc with GT and Q (Fig. 5).

### Reduced risk of liver damage by the combination treatment

**Fig. 6:**
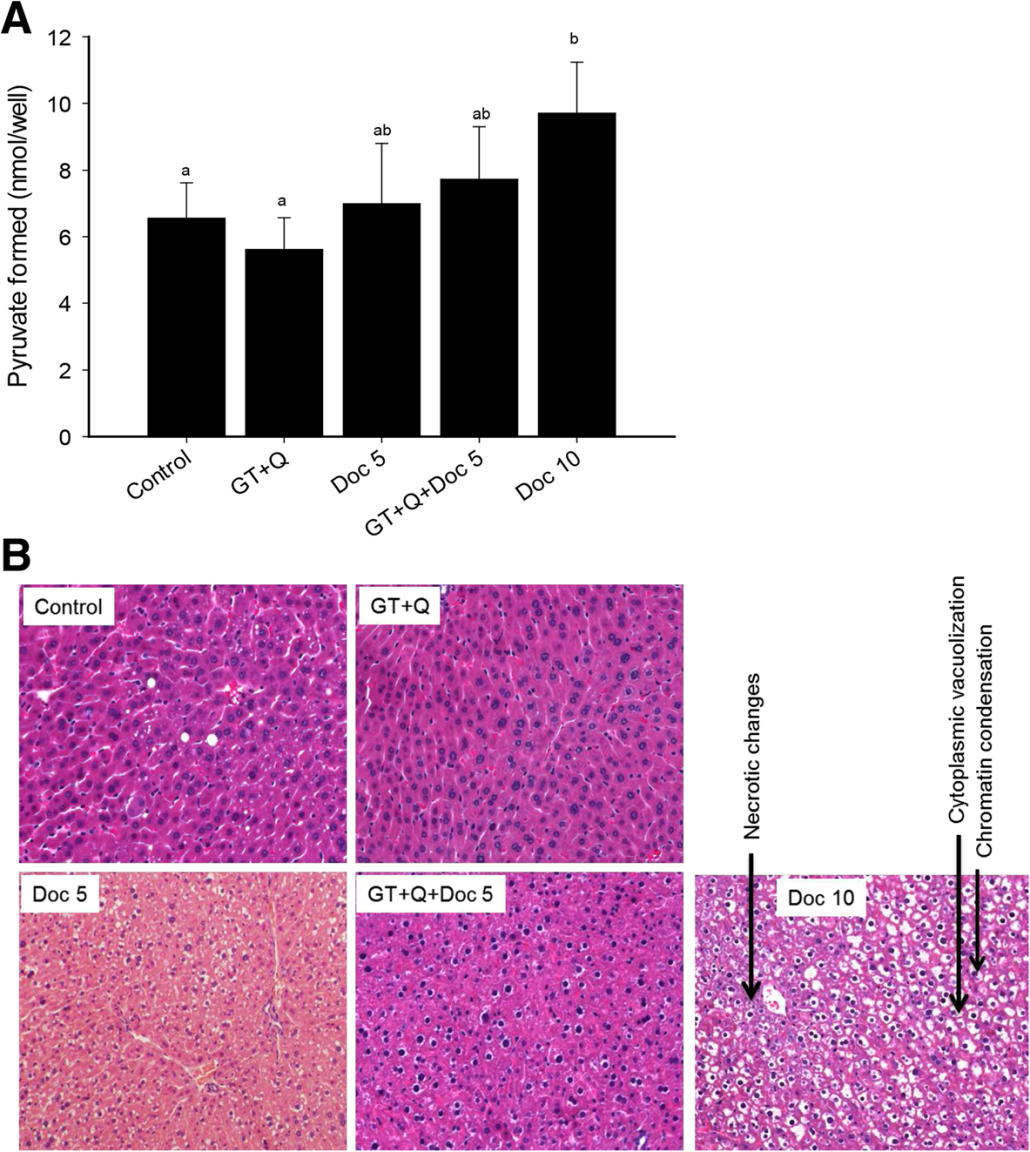
GT and Q reduced the risk of liver damage in enhancing the therapeutic effect of Doc. The liver condition was evaluated with both blood level of liver enzymes and liver pathological examination. Eight blood and liver samples were randomly selected from each group for this analysis. The blood alanine transaminase (ALT) level was determined by measuring its colorimetric product pyruvate using assay kit (**a**). Data are presented as mean ± SD. For the liver pathological examination, a section of liver tissue was fixed in 10 % phosphate buffered formalin and paraffin-embedded. Slides were cut and stained with H&E. The liver condition was graded based on the degree of inflammation, piecemeal or bridging necrosis, and representative images from each group were presented (**b**)

Liver toxicity is one of the common and dose-limiting side effects with Doc treatment. The evaluation of liver toxicity in this study was performed by measuring blood levels of the liver enzymes ALT and AST as well as by a pathological evaluation of liver tissues. There were no significant changes in blood ALT level with GT + Q, LD Doc, or their combination treatment compared to control, as indicated by the formation of pyruvate (Fig. 6a). However, a significantly elevated level of blood ALT was observed with the HD Doc treatment. The blood AST levels were not significantly different among groups (data not shown). The liver tissues were pathologically examined (Fig. 6b) and graded as following: 2.2 ± 0.5 (control), 2.2 ± 0.4 (GT + Q), 2.6 ± 0.8 (LD Doc), 2.7 ± 0.6 (GT + Q + LD Doc), and 3.2 ± 0.9 (HD Doc), with a significantly higher grade in HD Doc group compared to control or GT + Q group.

## Discussion

This study demonstrates that a combination of GT and Q with Doc significantly enhanced the potency of Doc by 2-fold in inhibition of PC-3 xenograft prostate tumor growth in SCID mice. The combined effect of the mixture was associated with increased apoptosis, decreased proliferation, and enhanced modulation of multiple signaling pathways. Tumors may have hundreds of gene mutations and dysfunctions and alternate signaling pathways may compensate for lack of function, thus a therapy targeting single or few pathways may not be effective and chemoresistance may develop soon. This is one of the major causes of the failure of most chemotherapy drugs including Doc [26]. Effort has been made to test the combinations of several other chemotherapy drugs such as vinorelbine and capecitabine with Doc [27]. However, no superiority to Doc/prednisone effect has been shown in phase III trials. In addition, these drug-drug combinations increase the challenge of adverse effects [27].

Natural compounds like GTPs and Q target multiple signaling pathways and events in anti-carcinogenesis, therefore in combination with chemotherapy drugs natural compounds may be able to provide a systemic control of the disease with enhanced therapeutic effect at lower doses of the drug. Previous studies showed that oral administration of GTPs in drinking water equivalent to a realistic dose for human consumption (4–6 cups of tea daily for an average adult human) significantly inhibited the growth of xenograft prostate tumors in mouse models and progression of prostate tumors in transgenic mice as demonstrated by our group and other investigators [10, 11]. Evidence from human studies is inconsistent, however, several recent clinical trials have demonstrated encouraging results [12, 13]. In a phase II clinical trial of tea in prostate cancer patients, we found that the consumption of GT at 6 cups a day for 1 month significantly decreased serum prostate-specific antigen (PSA) levels and nuclear NFκB staining in radical prostatectomy tissue as compared to control [14]. However, an analysis of GTPs in the prostate tissues revealed that about 50 % of EGCG was methylated into less active metabolites, which limits the anti-cancer activity of GT [15]. We found that the combined use of Q, a natural inhibitor of both catechol-O-methyltransferase (COMT) and multidrug resistance-associated proteins (MRPs), with GT significantly increased the bioavailability and cellular uptake of GTPs and decreased their methylation in vitro and in vivo, leading to a synergistically enhanced inhibition of xenograft prostate tumor growth in SCID mice [16–18]. Q itself has exhibited chemopreventive activities especially in prostate cancer through multiple mechanisms, including the induction of apoptosis and the inhibition of proliferation and insulin-like growth factor (IGF)-1 pathway [19–22].

The doses of GT and Q used in this study are physiologically relevant and safe for human consumption [28, 29]. The GT dose used is equivalent to the consumption of 5–6 cups of GT per day for an adult human, and Q 2 g per day for an adult. The same combination of GT and Q was able to significantly enhance the inhibition of tumor growth compared to GT or Q alone in SCID mice implanted with androgen-dependent LAPC-4 prostate cancer cells [14]. However, the administration of GT + Q without Doc was not significantly effective in inhibition of PC-3 xenograft tumor growth in the present study. These results are consistent with our previous in vitro observations that PC-3 cells were less sensitive to GT and Q compared to androgen-dependent LNCaP and LAPC-4 cells [30]. Treatment with LD Doc was initially effective, while tumors started to grow rapidly after the last dose of Doc and eventually tumors were not significantly smaller compared to control. However, a sustained inhibition of the tumor growth was achieved by the mixture of GT, Q, and LD Doc with a tumor growth inhibition comparable to that by a 2-fold higher dose of Doc treatment but without liver toxicity. Nevertheless, the treatment with the high dose Doc was associated with an increased risk of liver damage. These results indicate a promising clinical success of the mixture of GT, Q and Doc with enhanced therapeutic effect and reduced risk of side effects.

The combined effect of the mixture was associated with increased modulations on multiple signaling pathways and events. The mixture increased apoptosis of tumor cells as indicated by an increased ratio of Bax/Bcl-2 and downstream activation of caspase 7 along with increased cleavage of its substrate PARP1. A similar pattern of GT, Q and Doc in induction of apoptosis was also observed in our previous cell culture studies in PC-3 cells [8]. Reduced phosphorylation of Bad protein may contribute to tumor cell apoptosis in the present study, which allows the pro-apoptotic proteins Bax and Bak to aggregate and initiate apoptosis [31]. The decrease of IκB protein expression or phosphorylation in the present study suggests a potential role of the NFκB pathway in mediating the activity of the mixture. The tumor inhibitory effect through the inhibition of NFκB pathway was also demonstrated in previous studies [32]. In addition, our data demonstrated that a decrease in tissue protein content of a variety of growth factors including VEGF may play an important role in the reduction of tumor growth by GTP, Q and Doc [7, 33].

The present results also demonstrate the involvement of miRNA in the activity of GT, Q and Doc. The downregulation of the tumor suppressor miR-15a and miR-330 has been widely found in prostate tumors compared to normal tissues particularly in more advanced tumors, associated with tumor cell survival, proliferation and invasion [34, 35]. The cluster of miR-15a/miR16-1 was reported to target Bcl-2, CCND1 (encoding cyclin D1) and WNT3A, leading to tumor cell growth arrest and apoptosis [34]. The miR-330 has been shown to induce apoptosis possibly by targeting E2F1 and inhibit cell motility in PC-3 cells [35, 36]. In contrast, miR-19b, a potential oncomiR, was found to be involved in the suppression of the tumor suppressor phosphatase and tensin homologue (PTEN) and promote the proliferation of prostate cancer cells [37]. The increased miR-19b expression under Doc treatment in the present study may suggest a role of miR-19b in development of Doc resistance. The ability of GT and Q in combination with Doc to upregulate the expression of miR-15a and miR-330 and to balance miR-19b expression may partly contribute to the tumor inhibitory effect of the mixture in the present study.

## Conclusions

In summary, the combination of GT and Q with Doc significantly enhanced the potency of Doc in inhibition of PC-3 xenograft prostate tumor growth in SCID mice by targeting multiple events and signaling pathways involved in apoptosis and proliferation. This study provides a novel regimen to enhance the therapeutic effect of Doc in a less-toxic manner and reduce its risk of side effects in treatment of CRPC. These results warrant future clinical trial studies to confirm the combined effect of this mixture in humans.

## Abbreviations

ALT: alanine transaminase
AST: aspartate transaminase
CI: combination index
CRPC: castration-resistant prostate cancer
Doc: docetaxel
GT: green tea
GTPs: green tea polyphenols
EGCG: (-)-epigallocatechin-3-gallate
NFκB: nuclear factor-kappa B
PI3K: phosphatidylinositol 3-kinases
Q: quercetin
SCID: severe combined immunodeficiency
